# AI-driven ablative margin assessment after HCC radiofrequency ablation: a case-control feasibility study

**DOI:** 10.1186/s41747-026-00781-4

**Published:** 2026-09-08

**Authors:** Stefano Fogarollo, Gregor Laimer, Matthias Harders, Reto Bale

**Affiliations:** 1 Interactive Graphics and Simulation Group (IGS), Department of Computer Science, University of Innsbruck, Innsbruck, Austria; 2 Interventional Oncology/Stereotaxy and Robotics (SIP), Department of Radiology, Medical University Innsbruck, Innsbruck, Austria

**Keywords:** Artificial intelligence, Carcinoma (hepatocellular), Neoplasm recurrence (local), Radiofrequency ablation, Treatment outcome

## Abstract

**Objective:**

Thermal ablation of hepatocellular carcinoma (HCC) requires an adequate safety margin around the tumor to ensure treatment success. In this retrospective study, we evaluated an artificial intelligence (AI) pipeline for automatic safety margin assessment on computed tomography (CT) scans, with the goal of supporting radiologists in taking immediate clinical actions.

**Materials and methods:**

We included CT scans from patients with primary liver cancer treated at the local university hospital with radiofrequency ablation (RFA) between 2010 and 2022. The study group consisted of 43 randomly picked lesions with local tumor progression, with an average diameter of 35 mm [24–45] (median [interquartile range]). For comparison, we randomly selected 96 lesions showing no local tumor progression *via* propensity matching (diameter 28 mm [17–37]). To calculate the minimum ablative margin (MAM), we coregistered the intraprocedural patient scans with a recently proposed AI-based image registration model.

**Results:**

Overall, 139 HCC cases were evaluated, with an average follow-up interval of 25 months [8–42] (median, interquartile range). The calculated MAM was found to be a significant predictor of local tumor progression (odds ratio 0.42, 95% confidence interval 0.35–0.50, *p* = 0.001). Significant differences in local tumor protection rate were found between the lesions grouped by MAM (*p* < 0.001).

**Conclusion:**

Our AI-driven pipeline enabled fast and reliable assessment of the efficacy of RFA of HCC in relation to ablative margins.

**Relevance statement:**

The ablative margins have been automatically quantified using a novel method that combines AI-based segmentation with AI-driven image registration. By delivering robust margin quantification quickly, the proposed pipeline can help clinicians in assessing ablative margins intraoperatively.

**Key Points:**

- An AI-driven tool can rapidly and automatically measure the treatment margin after liver cancer ablation.
- Inadequate safety margins calculated with the proposed pipeline are associated with a higher risk of local tumor recurrence.
- A MAM of 2.50 mm suffices to lower the risk of local tumor progression to below 10%.

**Graphical Abstract:**

**Figure:**
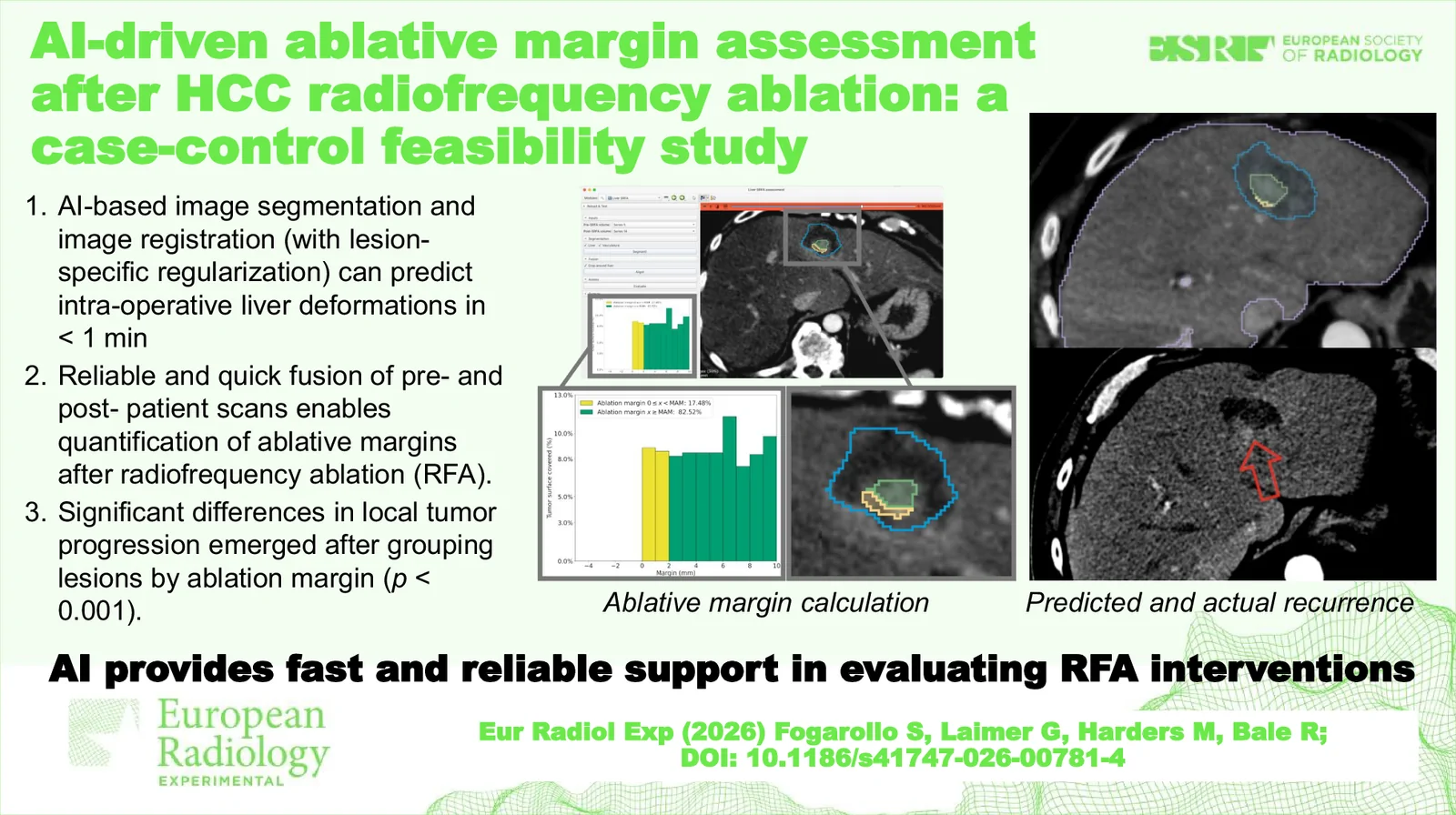

## Background

Hepatocellular carcinoma (HCC) is a major global health challenge, ranking as the sixth most common cancer and the fourth leading cause of cancer-related deaths worldwide [1]. Almost one million new cases were diagnosed in 2020, with a rising incidence in Europe and North America driven by increases in nonalcoholic fatty liver disease, hepatitis C, and other metabolic risk factors [2]. For patients with early-stage or small lesions (≤ 3 cm) [3], percutaneous thermal ablation has emerged as an effective, curative-intent treatment option. Furthermore, recent evidence from a multicenter randomized controlled trial [4] demonstrated that thermal ablation offers significantly fewer complications and shorter hospital stays compared to surgical resection in patients with HCC.

The success of thermal ablation treatments relies on achieving complete tumor necrosis with an adequate safety margin around the lesion [5]: the minimum ablative margin (MAM), defined as the shortest three-dimensional (3D) distance between the tumor boundary and the ablation zone [6], has been identified as an independent predictor of local tumor progression (LTP) in radiofrequency ablation (RFA) of HCC [7, 8]. Precise intraprocedural assessment of MAM is therefore essential for ensuring treatment efficacy and guiding immediate clinical decisions [6]. This requires fast and accurate coregistration of 3D patient scans, a process that is notoriously time-consuming and challenging to automate [9, 10]. The intra-procedural planning scan is done on the same day of the intervention, just before the ablation. This typically employs 1 mm slice thickness in the arterial phase. The intra-procedural evaluation scan is performed just after the ablation; this typically uses 3-mm slice thickness, in the portal venous phase. State-of-the-art finite-element method models have enabled quantification of ablative margins in secondary liver malignancies [11]. However, their clinical integration is limited by computational runtimes of several min [12], and the need for patient-specific tissue parameters for exact computations.

Recent advances in artificial intelligence (AI) [13–15] have demonstrated that image co-registration can be performed orders of magnitude faster than traditional techniques [16, 17]. For example, the work described in VoxelMorph [13] proposes training a convolutional neural network to predict the deformation field between two input images, achieving accuracy comparable to traditional methods. The image can be warped with a Spatial Transformer Network [18] on graphics processing units, reducing inference time from min to ms. Such dramatic speedups are crucial for clinical applications, such as intraprocedural image alignment, where minimizing delays for both patients and clinicians is essential.

Existing works have already applied AI-based registration for ablative margin analysis in HCC, but report computation times of > 3 min [19]. Furthermore, it is well known that translating AI methods to real-world registration tasks poses additional challenges, such as generalization [20] and plausibility [21]. While medical image segmentation is commonly applied to intraprocedural imaging to support automatic MAM assessment [12], there remains an unmet need for fast registration methods that can reliably align intraprocedural scans. Therefore, the goal of this study was to evaluate an AI-based deformable image registration method for quantitative margin assessment in patients with HCC undergoing computer tomography (CT)-guided RFA.

## Methods

This retrospective, single-center study was conducted with full approval from the Institutional Review Board (study 1094/2025) and in strict accordance with the Declaration of Helsinki. Written informed consent was obtained from all participants undergoing image-guided RFA. All cases included in this analysis were comprehensively reviewed, and treatment strategies were determined by consensus within multidisciplinary tumor board meetings comprising hepatologists, interventional radiologists, oncologists, and radiotherapists. The study was conducted entirely within a university hospital setting; no individuals from industry contributed to the study design, data collection, analysis, or interpretation. Furthermore, the investigation received no external funding or industry sponsorship, ensuring full scientific independence and objectivity.

### Study design

**Fig. 1:**
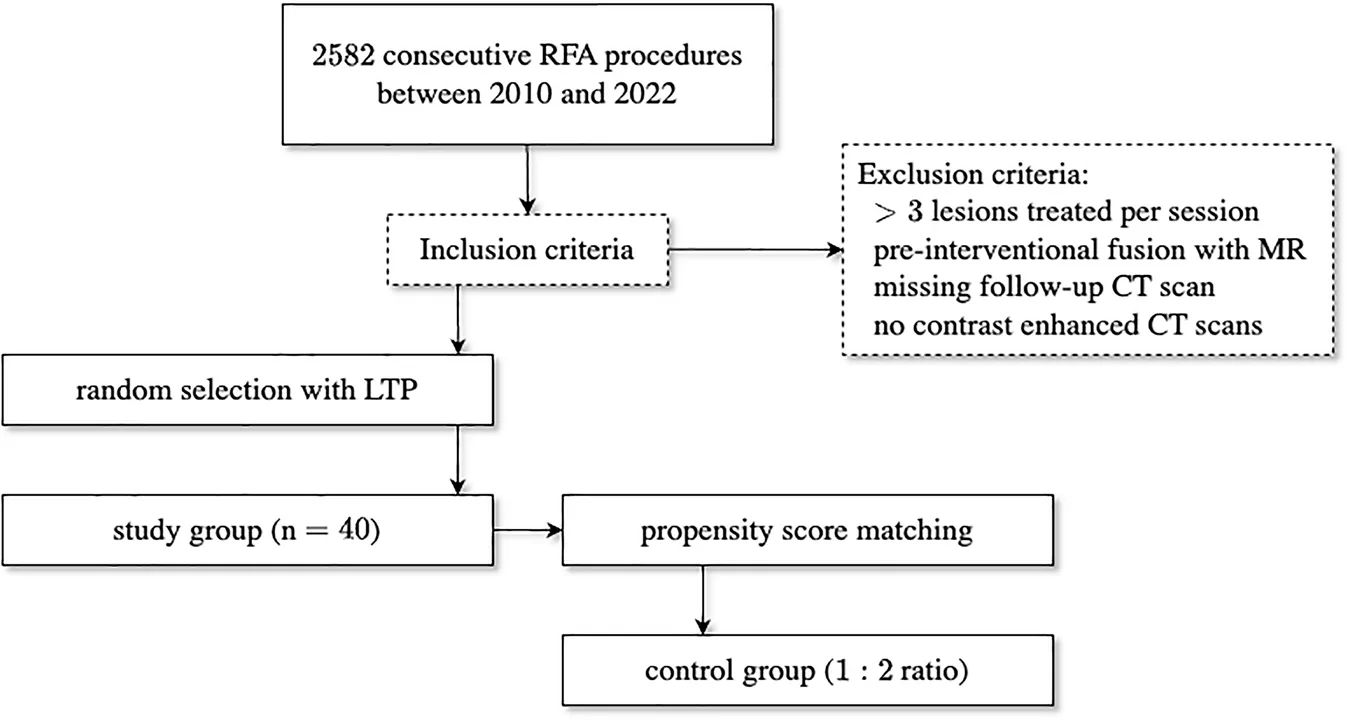
Flowchart of group assignment

We define LTP as the emergence of new tumor growth adjacent to the ablation zone [11], which is not visible at the follow-up immediately after the procedure. Between 2010 and 2022, 5,505 lesions in 1,627 patients were treated at the local University Hospital with 2,582 CT-guided RFA procedures. For this study, we first identified all eligible HCC lesions that showed LTP. We excluded procedures requiring intraprocedural magnetic resonance fusion or with more than three lesions treated per session. Confirmation of the absence of LTP required a minimum follow-up period of 24 months. However, when liver transplantation occurred within 24 months of the RFA session, follow-up ended, and any residual vital tissue was classified as LTP. Given the relatively low incidence of LTP (8.3% of all lesions), we elected to perform a case–control study on the lesions. First of all, we randomly assigned 40 lesions with LTP to the study group, selected from procedures with optimal imaging conditions (clearly defined liver and tumor boundaries) and without major complications [22]. In total, 8 procedures were excluded from the initial sampling because of unclear tumor boundaries due to the contrast agent. Then, we randomly selected lesions with no LTP using nearest neighbor propensity score matching with sex, age, number and tumor size, and liver function as matching variables, to achieve a case-control ratio of at least 1:2. If a lesion was included, we also included all the other lesions (if any) treated in the same procedure, as the LTP is influenced primarily by the local margin [5]. This also reflects real-world scenarios where multiple lesions may be treated and analyzed within a single intervention. Potential clustering effects arising from multiple lesions per patient or per procedure were addressed using mixed-effects models grouping at the patient and procedure level. This approach yielded a study cohort comprising 43 lesions with LTP and 96 without. The cohort size and case-control ratio follow the requirements to achieve a maximum marginal error of 10% at a 95% confidence interval [23]. All RFA procedures were performed with CT guidance by board-certified, experienced interventional radiologists, with patients under general anesthesia, using RFA (Cool-tip^TM^). We depict the flowchart of group assignment in Fig. 1. Detailed baseline characteristics of both groups are summarized in Table 1. Part of the data used in this study overlaps with a previous study [22]. The datasets analyzed during the current study are available from the corresponding author upon reasonable request.

**Table 1:** Statistics of included lesions (grouped at the patient level) in the study group (lesions with LTP) and control group (lesions without LTP)

Characteristics	Study group (*n* = 43)	Control group (*n* = 96)
Males	33	55
Females	6	8
Age (years)	70 [59, 74]	66 [58, 73]
Cirrhosis	81.4%	88.5%
Tumor size (diameter, mm)	35 [24, 45]	28 [17, 37]
Treated lesions	1 [1, 2]	1 [1, 2]
Follow-up (months)	25 [9, 41]	25 [8, 43]

Numbers are expressed as median and interquartile range

*LTP* Local tumor progression

Following the recent review of Paolucci et al [6], we designed a semi-automatic pipeline for MAM quantification as follows: first, we obtain tumor and ablation zone masks from the intraprocedural contrast-enhanced CT scans. Then, we co-register the scans, warp the masks, and finally, calculate the shortest 3D distance between the tumor and the warped ablation zone as the MAM.

### Liver, vasculature, tumor, and ablation zone segmentation

First of all, liver segmentation was performed using a mean ensemble approach that integrated three complementary pipelines: TotalSegmentator CT model [24], the Auto3DSeg from the MONAI framework [25], and a liver-oriented AI segmentation model [26]. Manual segmentations were obtained by an expert radiologist and independently verified by a senior radiologist to ensure reliability. The ensemble strategy was employed to minimize segmentation error in the final liver mask, following a strategy proposed in [27]. Then, the intraprocedural scans were cropped around the liver mask (with a 20-voxel margin per axis direction), thereby constraining the registration process to the relevant anatomical area. For the vasculature segmentation, a 3D nnUNet model [28] was trained on the VSNet dataset [29], which comprises 303 CT volumes containing the liver region. Tumor and ablation zone segmentations were performed using a 4-fold 3D nnUNet model [28], trained with active learning [24] on intraprocedural CT scans from 96 RFA procedures from the local University Hospital, not included in this study. This training involved three iterative cycles of model inference, expert refinement, and retraining. The entire segmentation pipeline enabled high-fidelity annotations of all 96 procedures within approximately one week.

### Deformable image registration

To coregister the intraprocedural scans, we trained a recent hybrid method for medical image registration [30] on the 96-procedure cohort (4 cross-folds). For this, we warped the intraprocedural CT scan immediately after ablation towards the CT scan taken at the start of the intervention. Then, around each lesion in the CT volume, we performed a fine-tuning step with instance optimization [15]. We imposed the loss from [31] as the image similarity penalty and the bending energy as the regularizer of the displacements [32]. We refer to Fig. 4a, b for an example of input images.

### MAM quantification

The deformation field was used to warp the ablation zone mask and overlay it on the intraprocedural volume coordinates. Due to volume resampling and the sub-millimeter precision required to measure the minimum margin, we converted both the tumor and the co-registered ablation area to a triangle mesh representation. This step avoids aliasing when calculating distances that are smaller than the voxel size. In the case of subcapsular lesions, we did not measure distances in points that are closer to the liver surface than a specified threshold (5 mm by default) [6]. Similarly, we excluded points adjacent to large vascular branches in the case of perivascular lesions [12]. The signed Euclidean distance from the boundary of the tumor *∂*T to the edge of the necrotic ablation zone *∂*A [12] was used as the margin.

Negative values correspond to points located outside the ablation area. Finally, the MAM was calculated according to [6], with values clamped to zero:

$$\begin{array}{l}{{{\rm{margin}}}}\left(x\right)=\left\{\begin{array}{cc}{\min }_{y\in \partial {{{\mathcal{A}}}}}x-y & x\;{{\in}}\;{{{\mathcal{A}}}}\\ {-\min }_{y\in \partial {{{\mathcal{A}}}}}x-y & x\;{{\notin}}\;{{{\mathcal{A}}}}\end{array}\right.\\ {{{\rm{MAM}}}}\left({{{\mathcal{T}}}} \right)=\max \left\{0,{\min }_{x\in \partial {{{\mathcal{T}}}}}{{{\rm{margin}}}}\left(x\right)\right\}.\end{array}$$

**Fig. 2:**
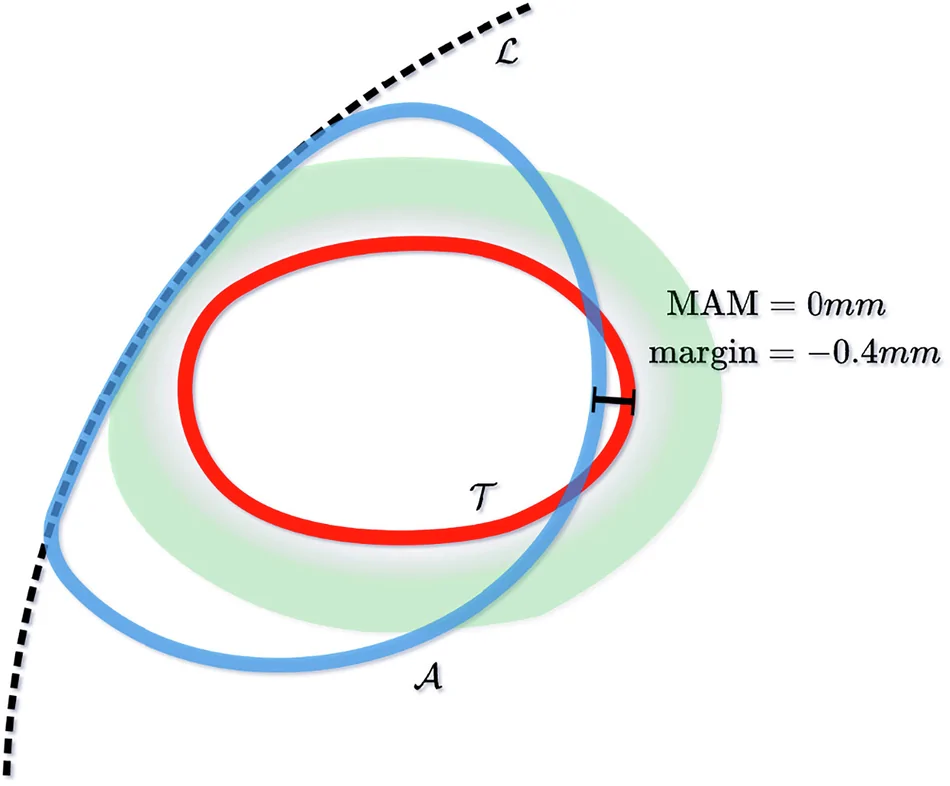
An example of MAM calculation on a subcapsular lesion T (border in red). The liver L edge (in dashed black) is used to limit the safety margin (in opaque green). In this case, the ablation area A (in blue) does not fully cover the safety margin around the tumor

We depict an illustrative example of MAM calculation in Fig. 2.

### User interface

To promote clinical usability and reproducibility, we developed a graphical extension for the 3D Slicer platform (v5.8) [33], which integrates the complete ablative margin quantification pipeline. The extension provides an interactive, step-by-step interface for executing all key components of the workflow, including segmentation of liver, tumor, and ablation zones; deformable image registration of intraprocedural CT scans; and quantitative measurement and visualization of the MAM.

**Fig. 3:**
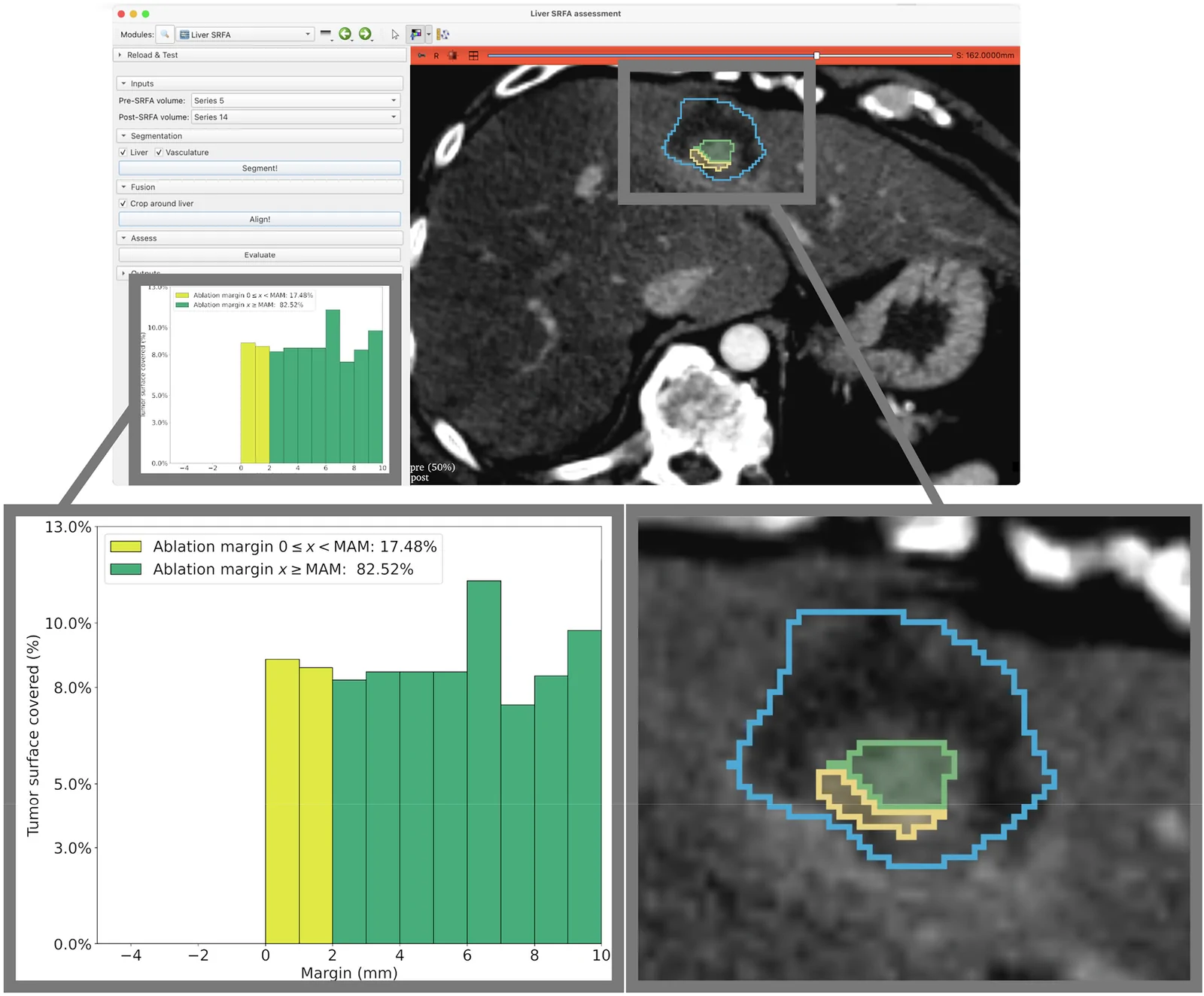
A screenshot of the proposed 3D Slicer extension. Here, we used the blending option to visualize the intraprocedural computed tomography (CT) scans after registration, both with 50% opacity. The outline of the ablation zone is colored in blue. We used yellow and green to mark areas of the tumor with a margin between 0 and 2 mm, and ≥ 2 mm, respectively [see reference [11]]

Upon loading the relevant patient scans, users can initiate segmentation using local models (TotalSegmentator [24], Auto3DSeg [25], and the pre-trained nnUNet models [28]), with optional manual review and correction via the Slicer Segment Editor module. The registration module is then executed via a single button, automatically performing deformable alignment and fine-tuning around each segmented lesion of interest. Margin computation is fully automated, and results are displayed as both numerical summaries (minimum margin in millimeters) and color-coded 3D overlays showing distance-to-boundary values across the tumor surface. The interface enables the visualization of both the original and registered scans in axial, sagittal, and coronal views, alongside synchronized 3D renderings of the tumor and ablation volumes. We visualize a screenshot of the extension in Fig. 3. Key steps, including segmentation results, registration displacement fields, and margin heatmaps, are saved automatically for reproducibility and quality control. This interface was designed in close consultation with interventional radiologists to support both retrospective analysis and potential real-time intraprocedural use, prioritizing automation, interpretability, and minimal user interaction. The extension will be made publicly available upon acceptance of the manuscript.

### Statistical analysis

We used Python 3.10 and the psmpy package for the study and control group selection. The Kaplan–Meier estimator was used to assess differences between groups (using the log-rank test for comparison). All statistical analyses were performed retrospectively and with statsmodels v0.14.4, scipy v1.13.1, and R 4.5.2. A *p* < 0.05 was considered statistically significant.

## Results

### Patient characteristics and ablation outcomes

Overall, this study included 139 lesions, of which 43 lesions had LTP and 96 without, spanning 100 interventions and 96 patients (14 females and 82 males) with a median age at procedure time of 67 [interquartile range 58–74] years. Over a median follow-up time of 25 [8–42] months, LTP was noted in 43 of the 139 ablated lesions. We report quantitative results grouped by lesion group in Table 2.

**Table 2:** Computational results grouped by study group (lesions with LTP) and control group (lesions without LTP)

Parameter	Study group (*n* = 43)	Control group (*n* = 96)
Liver ASSD (mm)	0.93 ± 0.43	0.86 ± 0.31
Liver HD95 (mm)	4.08 ± 4.66	3.49 ± 3.17
Margin (mm)	0.4 ± 0.8	2.0 ± 2.2

Numbers are expressed as mean and standard deviation

*ASSD* Average symmetric surface distance, *HD95* 95th percentile of the Hausdorff distance, *LTP* Local tumor progression

### Liver, vasculature, tumor, and ablation zone segmentation

The ensemble liver segmentation pipeline yielded high-fidelity predictions on the CT volumes examined, in agreement with expert manual contours: no further correction was needed. Vascular segmentation on the VSNet cohort (*n* = 303) achieved a mean Dice coefficient [34] of 0.77 ± 0.06 (mean ± standard deviation), with an average symmetric surface distance (ASSD) of 1.94 ± 0.94 and a 95th percentile of the Hausdorff distance (HD95) of 13.87 ± 8.25, demonstrating robust delineation across variable slice thicknesses [35]. The four-fold 3D nnUNet trained with active learning on the 96-scan validation set produced, respectively, for the lesion and the ablation zone, mean Dice scores of 0.61 ± 0.36 and 0.82 ± 0.21, 4.79 ± 8.39 mm and 4.14 ± 6.03 mm for ASSD, 20.04 ± 28.07 mm and 24.55 ± 39.31 mm for HD95. The average inference time per volume was 28.46 ± 7.15, 8.66 ± 4.82, 7.28 ± 3.78, 9.82 ± 5.01 s, respectively for liver, vasculature, tumor, and ablation masks, underscoring the pipeline’s potential for intraprocedural clinical integration.

### Deformable image registration

**Fig. 4:**
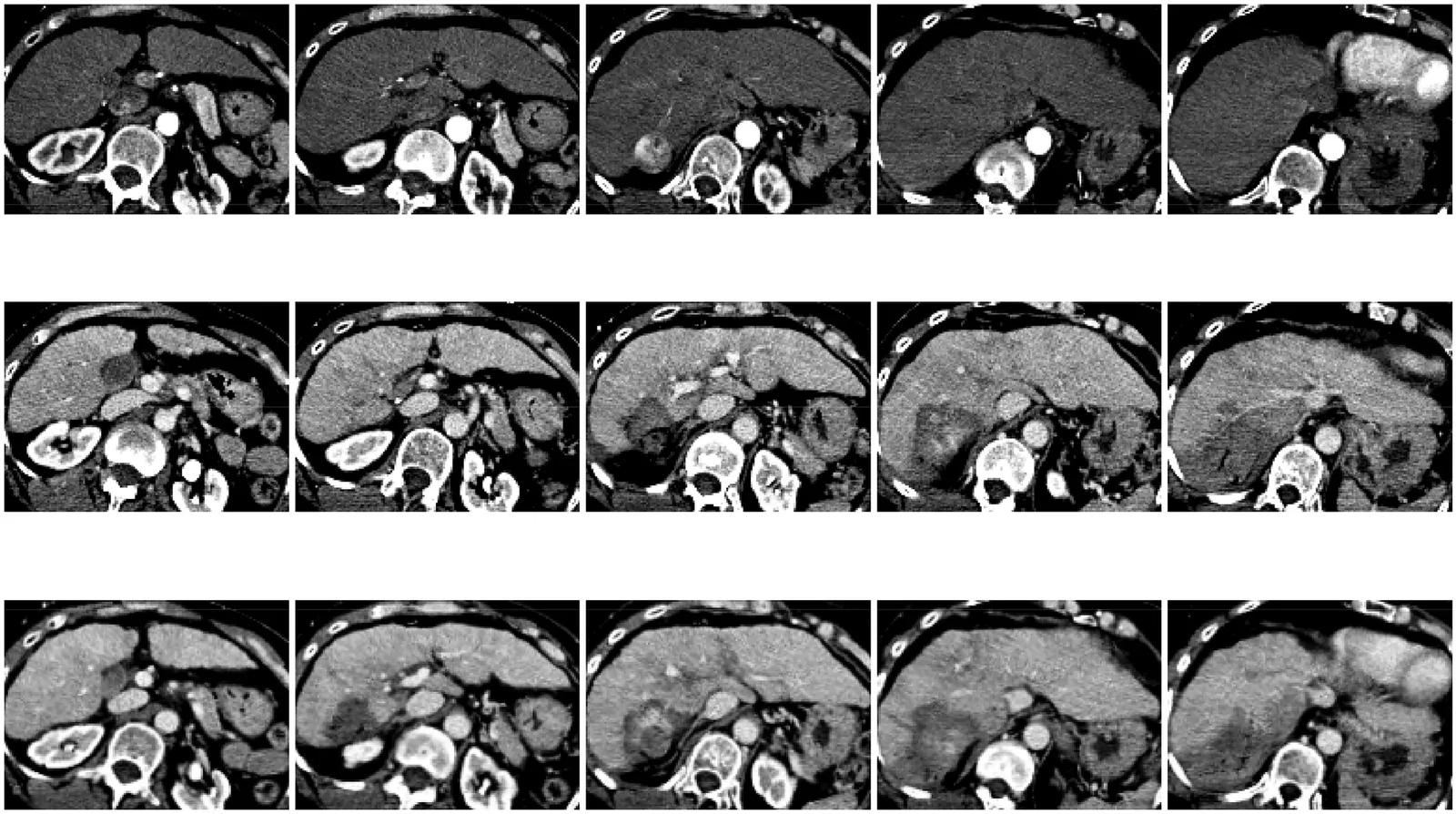
Intraprocedural image of a randomly picked procedure, performed in 2015. Top: intraprocedural imaging just before the intervention (fixed). Middle row: intraprocedural imaging just after the intervention (moving), after rigid alignment. Bottom: result of warping the moving image with the dense deformation field predicted with our pipeline. Note the improvement in vasculature alignment despite the poor contrast

We report liver surface registration accuracy using two key metrics: the ASSD and the HD95, both expressed in mm. The average ASSD was 0.87 ± 0.34 mm, while the HD95 measured 3.55 ± 3.13 mm. Both the global registration method [30], and the lesion-specific fine-tuning step [15], operate within seconds: the first required a mean of 1.63 ± 0.27 s per CT volume, while the second required 7.62 ± 4.54 s per lesion. In total, the end-to-end registration pipeline completed in 12.22 ± 6.58 s per procedure. Importantly, the entire registration workflow was fully automated, with no manual modifications applied to the displacement fields at any stage of the analysis. All cases were successfully processed without the need for operator intervention, and none were excluded due to suboptimal registration quality. In Fig. 4, we depict an example of co-registration using our proposed pipeline.

### MAM quantification

**Fig. 5:**
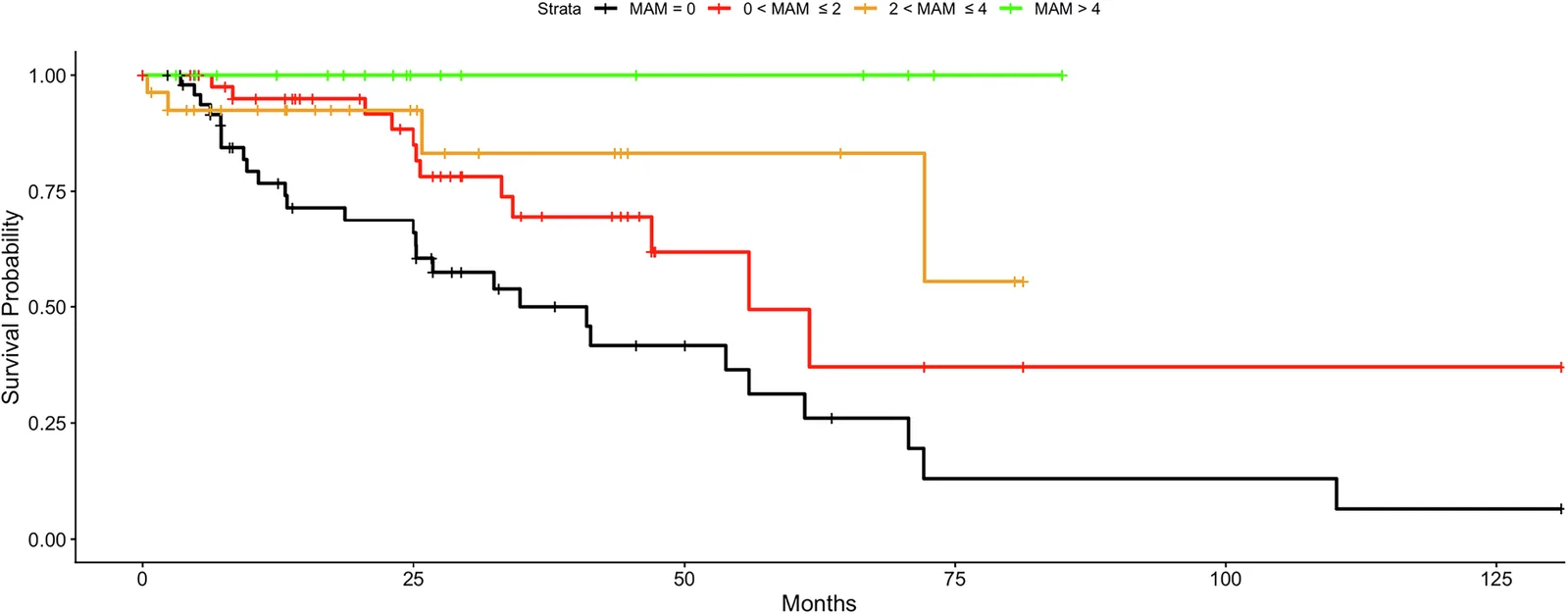
Kaplan–Meier curves stratified by margin category

Among the 139 ablated lesions, 50 (36%) demonstrated a MAM = 0 mm, 44 (32%) showed a MAM > 0 and < 2 mm, and 45 (32%) had a MAM ≥ 2 mm. LTP was observed in 27 lesions (54%) with a MAM of 0 mm, and 12 lesions (27%) with a MAM between > 0 and < 2 mm. Four (9%) lesions with MAM > 2 mm showed LTP. No LTP has been detected in lesions showing a MAM > 4 mm. We plot the Kaplan-Meier curves on survival probability stratified by margin category in Fig. 5. Finally, we found significant differences among groups (χ^2^ = 19.5, *p* < 0.001).

### Analysis of risk factors in LTP

To mitigate the clustering effect from same-intervention lesions, we fit two generalized linear mixed-effects models to predict the local recurrence, first grouping by patient, and then by procedure. The MAM was the only factor showing a statistically significant correlation with local recurrence, both when grouping by patient (odds ratio = 0.43, 95% confidence interval 0.35–0.51, *p* < 0.001) and by procedure (odds ratio = 0.42, 95% confidence interval 0.35–0.50, *p* = 0.001). When grouping by patient, the *p*-values associated with the other factors were 0.655, 0.994, 0.986, 0.604, 0.984 for patient’s sex and age, lesion size, liver ASSD and liver HD95, respectively. When grouping by procedure, the *p*-values were 0.654, 0.994, 0.986, 0.562, 0.986.

**Fig. 6:**
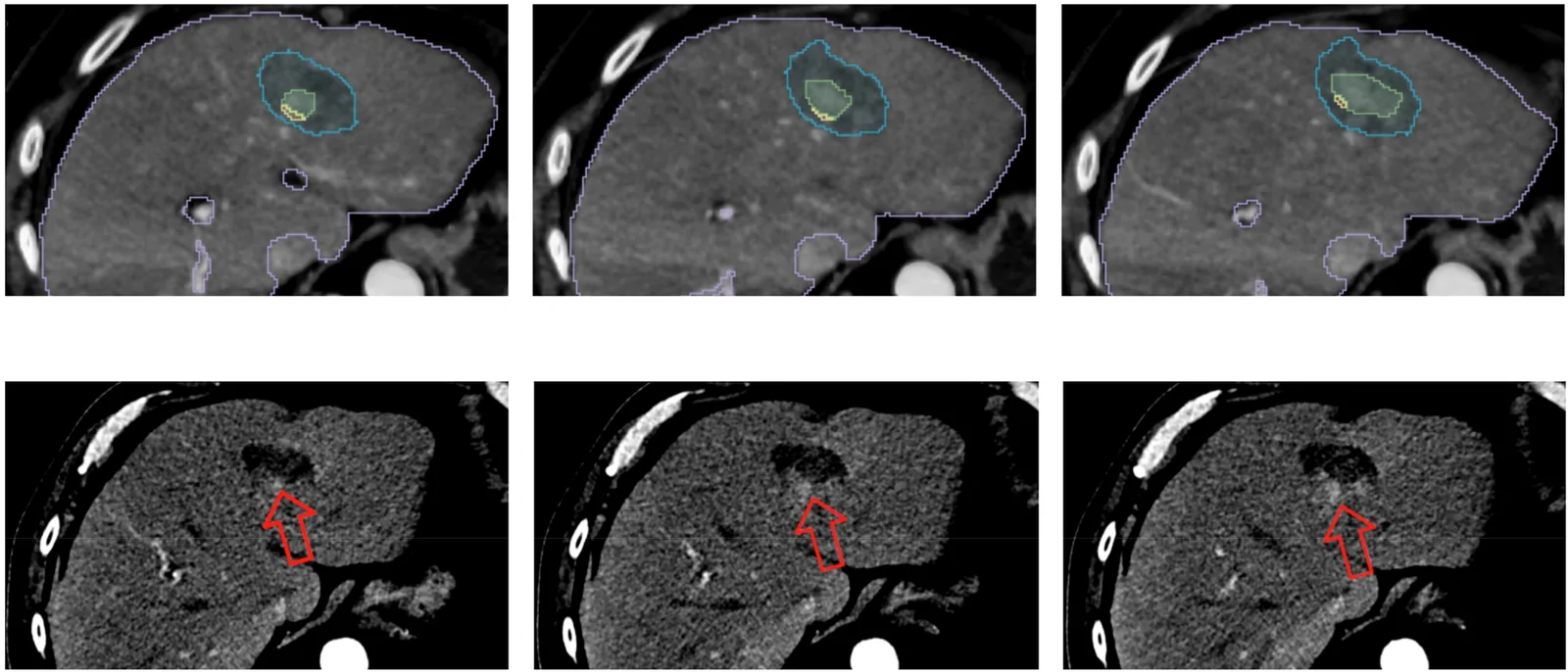
Lesion at risk of developing recurrence (marked in yellow in the top figure), and recurrence visible from the follow-up scan (28 months later). Top row: overlay of pre-interventional arterial CT scan (50%) and coregistered post-interventional portal venous scan (50%) with segmentations and margins (consecutive slices). The liver contour is outlined in purple, while the ablation contour is in blue. Points inside the lesion are colored based on the margin: in green, the points with a sufficient margin, and in yellow, the points at risk of developing recurrence. Bottom row: consecutive slices from the follow-up scan in arterial phase. The location of recurrence is indicated with a red arrow

To characterize the relationship between MAM and the 2-year risk of LTP, we used the model grouped by procedure. A MAM of 2.50 mm was sufficient to reduce the risk of LTP below 10%. Following [11], the A0 margin, defined as the smallest margin yielding a 2-year LTP risk of less than 5%, was calculated to be 3.50 mm. Finally, the point of diminishing returns, at which each additional 1 mm increase in MAM yielded less than 1% absolute reduction in LTP risk, was identified at 4.70 mm. We depict in Fig. 6 the spatial correlation between the predicted LTP and the actual recurrence, as visible in the follow-up scan.

## Discussion

In this feasibility study, we developed and evaluated a fully automated AI pipeline for quantitative measurement of the MAM in CT-guided RFA of HCC. Our pipeline integrated automated liver, vasculature, tumor, and treatment area segmentation, deformable registration, and volumetric margin quantification. While the relationship between insufficient margin and LTP is well known in the clinical literature [5], the main contribution of this study lies in the technical development of a fast and robust MAM quantification pipeline.

Previous margin-assessment methods have relied on manual or semi-automated techniques [10]. Traditionally, radiologists have evaluated margins by visually inspecting paired CT scans or using rigid image fusion software [5]. However, purely visual assessment is notoriously imprecise due to inter-reader variability [9]. Indeed, a systematic review found that in approximately one-third of studies, radiologists’ visual estimates of margin overestimated the true (quantitatively measured) margin [10]. Deformable registration approaches can capture organ deformation and yield higher accuracy [36]. However, these methods require complex computations and tuning of boundary conditions, making them time-consuming and impractical for routine clinical use. In the pipeline proposed in this study, we narrow the gap between speed and accuracy: automatic AI-based segmentations combined with AI-driven multi-step image registration help achieve trustworthy results in about 1 min (54.22 ± 10.67 s for image segmentation and 12.22 ± 6.58 s for image registration per procedure), a significant speed-up compared to traditional approaches [37].

We note that no procedure was excluded due to registration quality, highlighting the robustness and generalization of the proposed method. Fast identification of insufficient margins could directly inform the operator to apply additional ablation immediately, streamlining the clinical workflows and improving patient outcomes. By providing an objective and quantitative margin measurement, the pipeline could augment the clinician’s judgment and reduce the inter-operator variability in assessing the MAM.

The following limitations of this study warrant discussion. First of all, the data derive from a single center using a retrospective, case-control design. This artificial sampling inflates the proportion of LTP events relative to usual practice, and thus the reported accuracy may not reflect a typical prospective cohort [8]. Second, the retrospective design introduces inherent risks of selection bias. Specifically, the availability and quality of imaging data (particularly the absence of contrast-enhanced CT scans) may have influenced case inclusion. Nonetheless, it is essential to note that the AI-based segmentation and registration models employed in this study were designed to remain robust even in challenging imaging conditions with poor contrast, thereby mitigating some of the limitations associated with data heterogeneity. Finally, we did not benchmark the proposed margin quantification method against other commercially available deformable registration tools. Comparative validation studies are therefore needed to further establish the accuracy and clinical utility of our approach in routine practice. A prospective multi-center validation study should be undertaken to confirm these findings in a broader patient population and across different institutions and workflows. Integration of the pipeline into ablation treatment planning systems or real-time navigation platforms could enable intraprocedural use, allowing for immediate feedback. Expansion to other tumor types (such as colorectal liver metastases) and other ablation modalities (microwave, cryoablation) is also feasible.

In conclusion, a significant correlation has been found between the MAM calculated using the proposed pipeline and LTP after RFA. The MAM has been quantified using a fast and reliable method that combines AI-based segmentation with AI-driven registration models. By delivering accurate, automated, and anatomically consistent margin quantification in under a minute, this pipeline has the potential to support ablation treatment evaluation and assist radiologists in verifying complete tumor coverage in clinical practice.

## Supplementary information

**Additional File 1: Figure S1:** Co-registration overlay and lesion at risk after a RFA procedure on a 74 year old woman (a), and follow-up scan 12 months later (b). **Figure S2:** Co-registration overlay and lesion at risk after a RFA procedure on a 82 year old man (a), and follow-up scan 24 months later (b). **Figure S3:** Co-registration overlay and lesion at risk after a RFA procedure on a 76 year old man (a), and follow-up scan 13 months later (b). **Figure S4:** Co-registration overlay and lesion at risk after a RFA procedure on a 74 year old man (a), and follow-up scan 6 months later (b). **Figure S5:** Co-registration overlay and lesion at risk after a RFA procedure on a 74 year old man (a), and follow-up scan 26 months later (b). **Figure S6:** Co-registration overlay and lesion at risk after a RFA procedure on a 71 year old man (a), and follow-up scan 11 months later (b).

## Data Availability

The datasets used and/or analyzed during the current study are available from the corresponding author on reasonable request.

## Abbreviations

3D: Three-dimensional
AI: Artificial intelligence
ASSD: Average symmetric surface distance
CT: Computed tomography
HCC: Hepatocellular carcinoma
HD95: 95th percentile of the Hausdorff distance
LTP: Local tumor progression
MAM: Minimum ablative margin
RFA: Radiofrequency ablation
