# Supplementary material for: AI-driven ablative margin assessment after HCC radiofrequency ablation: a case-control feasibility study
Source: Eur Radiol Exp. 2026 Sep 8;10:117. doi: 10.1186/s41747-026-00781-4 (PMC13554041; doi:10.1186/s41747-026-00781-4)
Supplement: Supplementary file 1 [file 41747_2026_781_MOESM1_ESM.pdf]

AI-driven ablative margin assessment after HCC radiofrequency ablation: a case-control feasibility study

Supplementary Information

In the following, we qualitatively compare the regions at risk of developing local recurrence as predicted by our method, with the actual recurrence sites observed in follow-up patient scans taken several. First, we overlay the pre-interventional CT scan (arterial phase, 50%) with the co-registered post-interventional CT scan (portal venous phase, 50%). We contour the treatment area in blue and visualize the related tumor risk map: in green the tumor points with a sufficient margin, and in yellow the points at risk of developing recurrence. Then, we indicate the location of the actual recurrence with a red arrow.

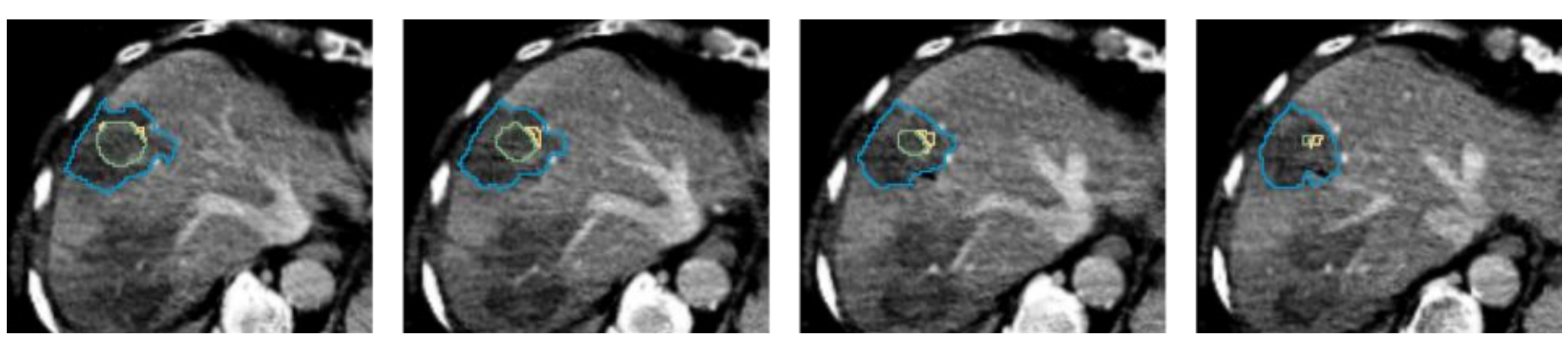

(a) Intervention done on 16 May, 2014.

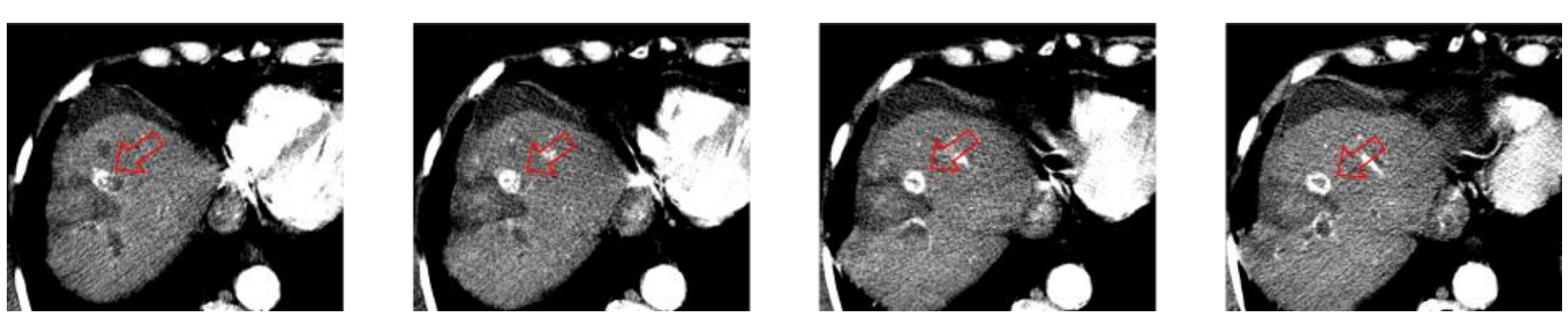

(b) Consecutive slices from the follow-up CT scan on 22 May 2015.

Figure S1: Co-registration overlay and lesion at risk after a RFA procedure on a 74 year old woman (a), and follow-up scan 12 months later (b).

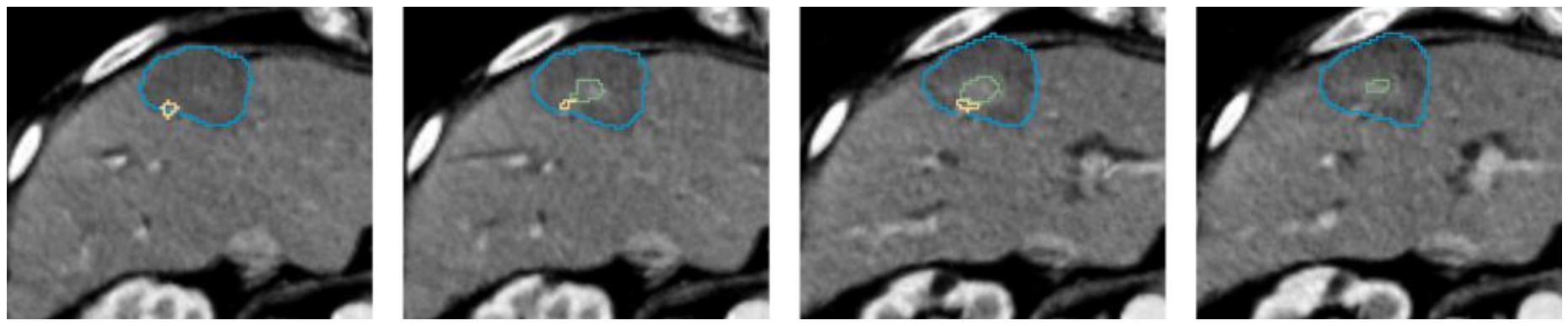

(a) Intervention done on 15 February 2022

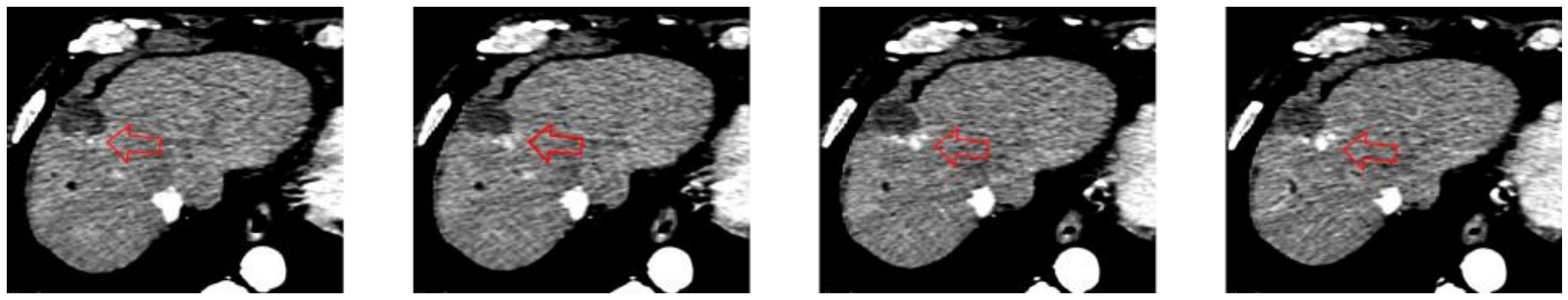

(b) Consecutive slices from the follow-up CT scan on 15 February 2024.

Figure S2: Co-registration overlay and lesion at risk after a RFA procedure on a 82 year old man (a), and follow-up scan 24 months later (b).

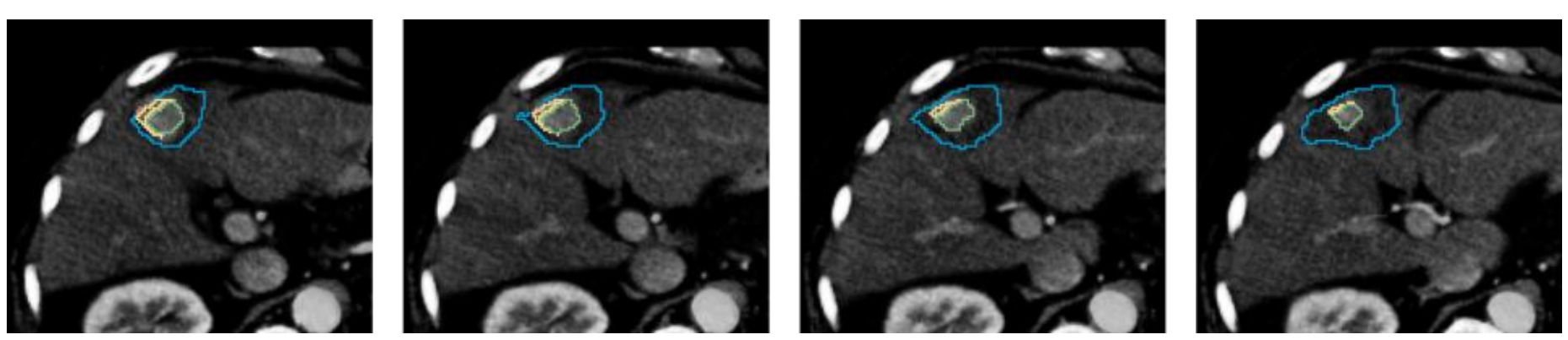

(a) Intervention done on 13 April 2016.

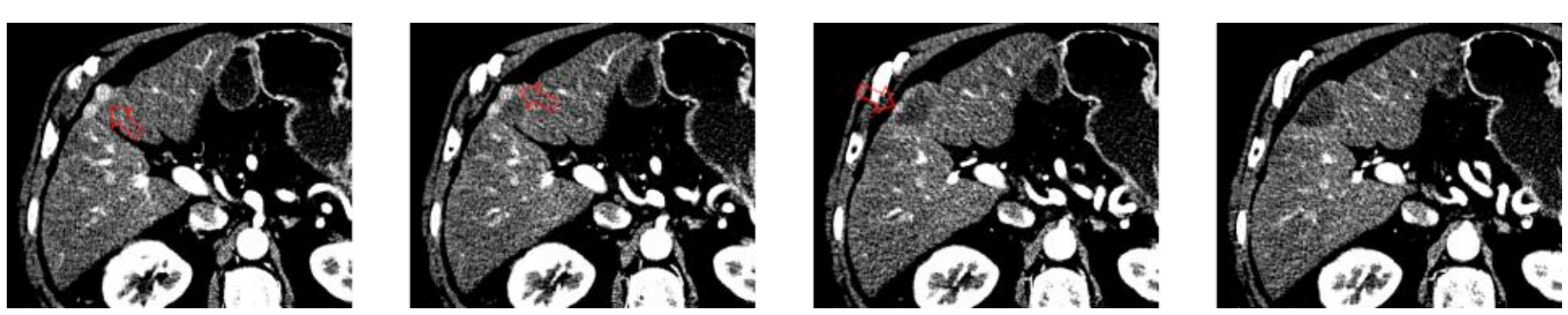

(b) Consecutive slices from the follow-up CT scan on 17 May 2017.

Figure S3: Co-registration overlay and lesion at risk after a RFA procedure on a 76 year old man (a), and follow-up scan 13 months later (b).

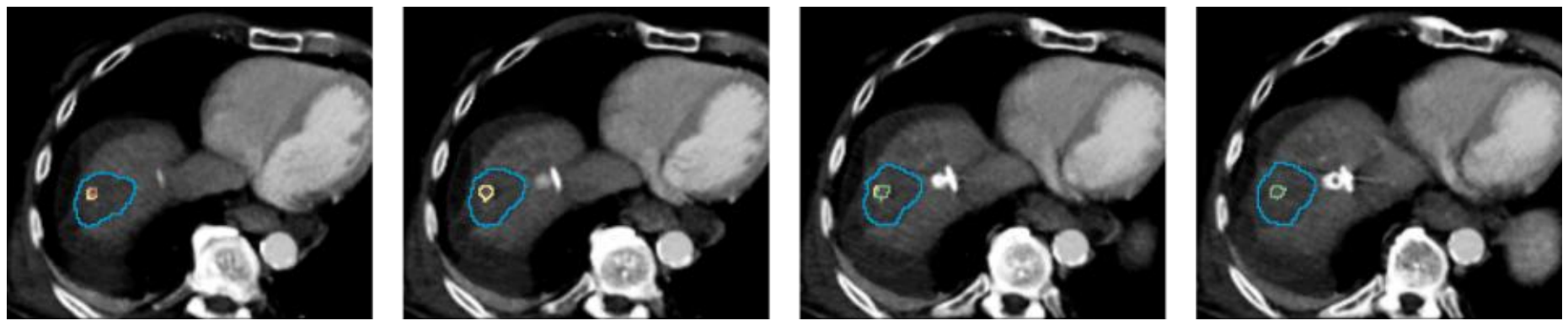

(a) Intervention done on 25 September 2019.

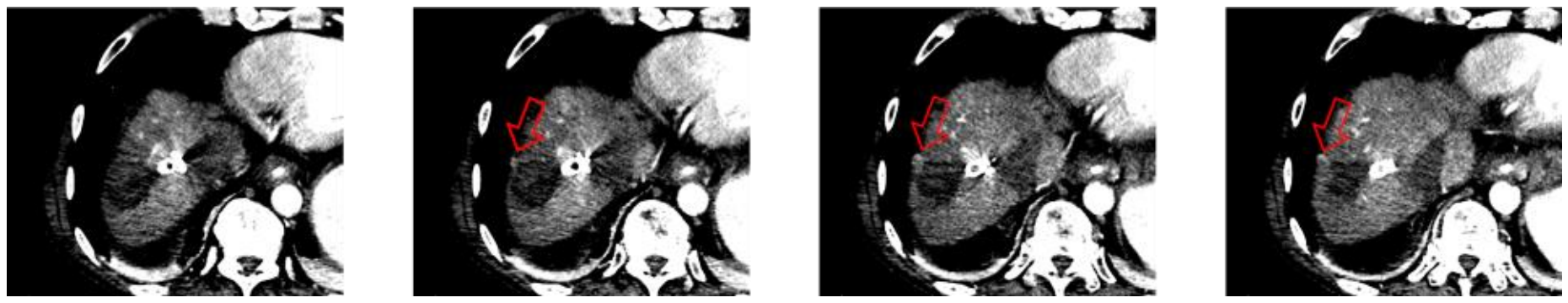

(b) Consecutive slices from the follow-up CT scan on 3 April 2020.

Figure S4: Co-registration overlay and lesion at risk after a RFA procedure on a 74 year old man (a), and follow-up scan 6 months later (b).

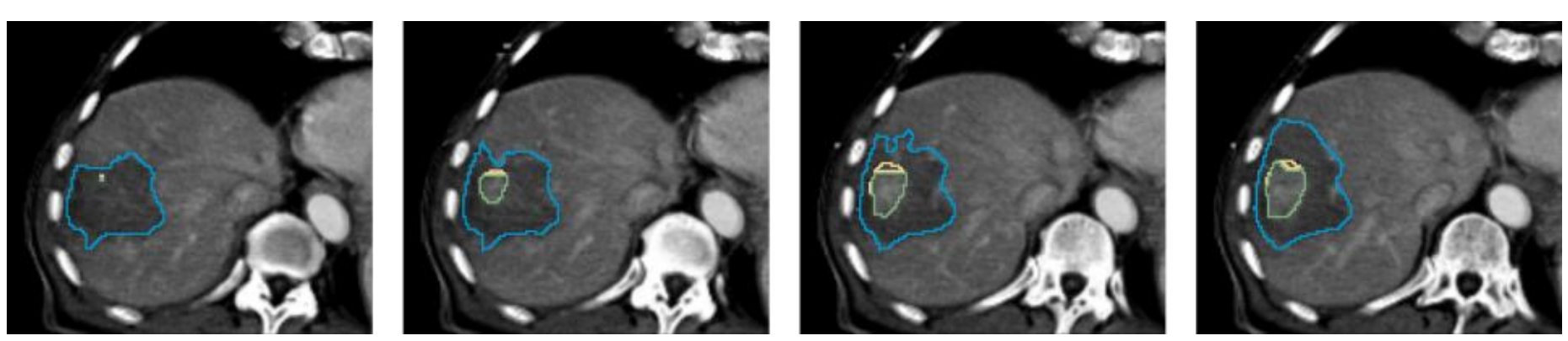

(a) Intervention done on 15 May 2014.

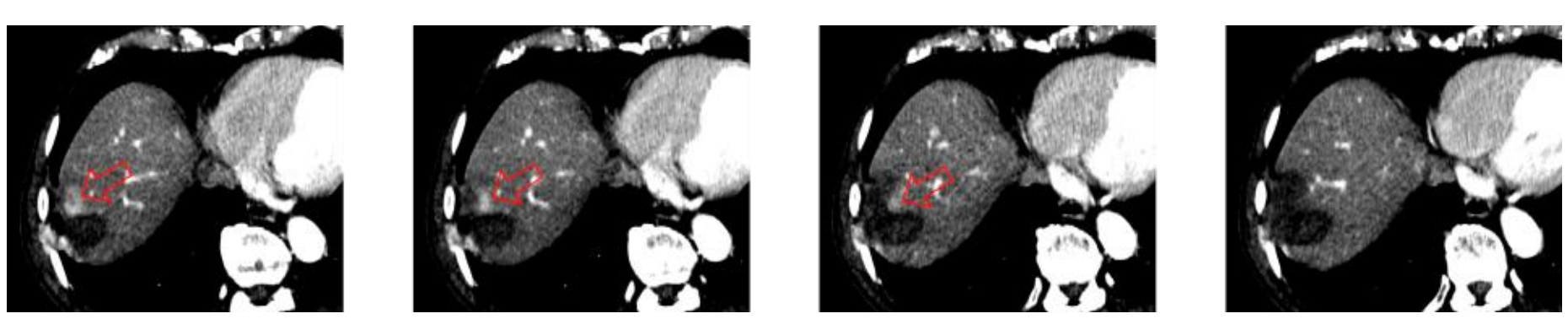

(b) Consecutive slices from the follow-up CT scan on 22 June 2016.

Figure S5: Co-registration overlay and lesion at risk after a RFA procedure on a 74 year old man (a), and follow-up scan 26 months later (b).

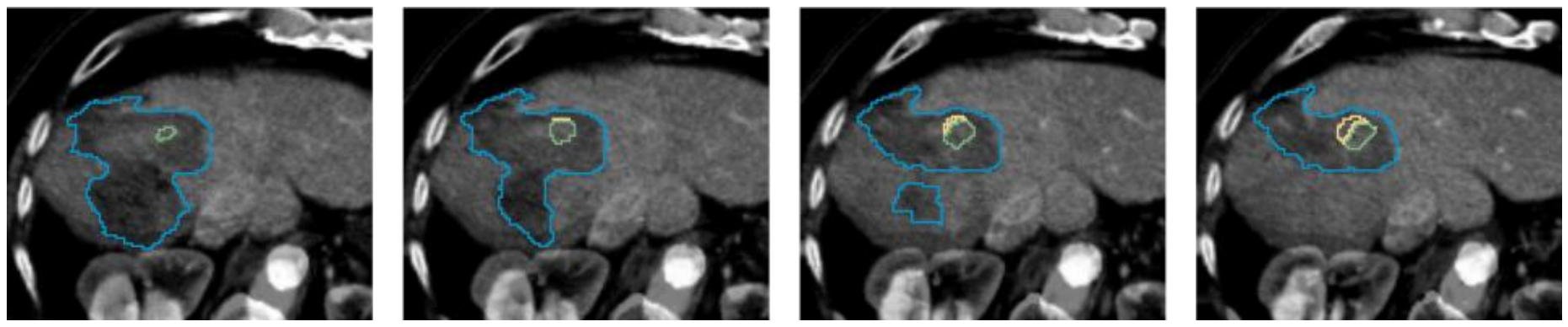

(a) Intervention done on 19 November 2015.

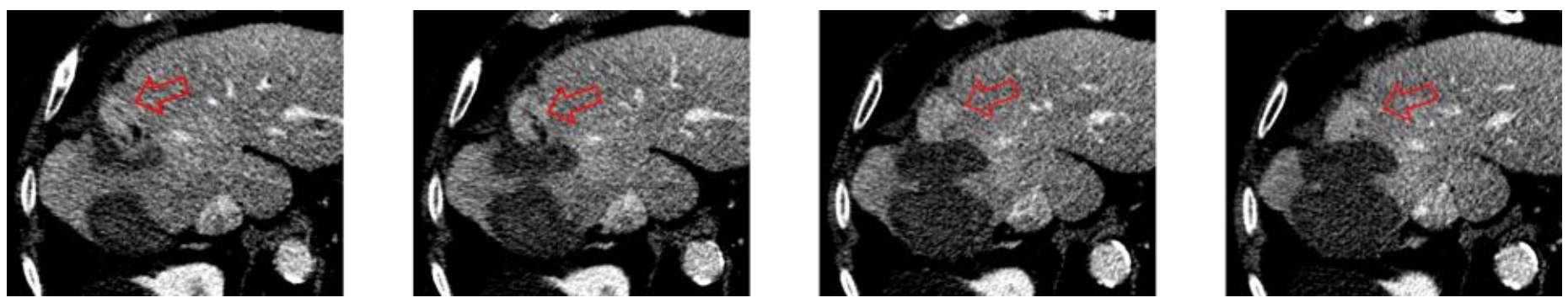

(b) Consecutive slices from the follow-up CT scan on 4 October 2016.

Figure S6: Co-registration overlay and lesion at risk after a RFA procedure on a 71 year old man (a), and follow-up scan 11 months later (b).
